# Isothermal Amplification of Long, Discrete DNA Fragments Facilitated by Single-Stranded Binding Protein

**DOI:** 10.1038/s41598-017-09063-x

**Published:** 2017-08-17

**Authors:** Yinhua Zhang, Nathan A. Tanner

**Affiliations:** New England Biolabs, 240 County Road, Ipswich, MA 01938 USA

## Abstract

Isothermal amplification methods for detection of DNA and RNA targets have expanded significantly in recent years, promising a new wave of simple and rapid molecular diagnostics. Current isothermal methods result in the generation of short fragments (<150 base pairs) or highly branched long DNA products. Here we report the amplification of discrete target fragments of several kilobases at 37 °C from both double- and single-stranded circular template DNA using specific primer pairs. In contrast to existing methods, this amplification requires only the single-stranded DNA-binding protein gp32 from bacteriophage T4 and a strand-displacing DNA polymerase. In addition to the discrete amplicon products, this method also produces higher molecular weight products consisting of multiple repeated copies of the amplicon and template DNA. We demonstrate that two features of gp32 enable this amplification: a facilitation of primer strand invasion into double-stranded DNA, and a suppression of non-homologous primer annealing and nonspecific amplification. The ability presented here to produce long, discrete DNA products in an isothermal reaction extends the scope of isothermal amplification to enable more useful applications of these promising methods.

## Introduction

Much of modern biotechnology and molecular biology relies on the ability to manipulate a specific nucleic acid target, but in order to efficiently detect, read, or use that target sequence, it must be available at sufficient quantities in the application. Most methods involving DNA or RNA start with the molecules of interest at concentrations far below the necessary amount, and accordingly must be increased through amplification. DNA amplification can be performed using a variety of strategies, most commonly the polymerase chain reaction (PCR) which is enabled by cycles of thermal denaturation of double-stranded DNA (dsDNA). While routine and flexible, PCR is limited in application by its requirement for instruments capable of thermal control and cycling between high temperatures for dsDNA denaturation and lower temperatures for primer extension by the DNA polymerase. Additionally, the degree of amplification is intrinsically limited by the cycling of the reaction: each cycle can at most produce a doubling of the DNA. Recent years have seen growing adoption of methods that amplify DNA or RNA at a single temperature, accordingly referred to as “isothermal” amplification. These methods require simpler instrumentation, using only a single, moderate temperature. Additionally, isothermal methods can provide extremely rapid results, as fast as 10 minutes, and can produce a large amount of DNA, enabling simple, visual detection of amplification^1,2^.

Each isothermal technique relies on enzymatic activities or primer design to bypass the need for thermal denaturation of dsDNA. Approaches vary, but generally strand displacement activity by a DNA polymerase (typically the large fragment of *Bsu, Bst*, and *E. coli* DNA Polymerase I, or phi29 DNA polymerase) separates dsDNA after initiation at a primer, with the initiation step proving the key limit to speed and efficiency of an isothermal reaction. Strategies to facilitate initiation provide the main variance among isothermal methods. For example, initiation approaches include: creation of nicks by a nicking enzyme as in strand displacement amplification (SDA)^3^ and nicking enzyme amplification reaction (NEAR)^4^; facilitated strand invasion as in strand invasion-based amplification (SIBA)^5^, recombinase polymerase amplification (RPA)^6^ and helicase-dependent amplification (HDA)^7^; or thermodynamic invasion and annealing as in the initiating first round of SDA, NEAR, loop-mediated isothermal amplification (LAMP)^8^, multiple displacement amplification (MDA) and rolling circle amplification (RCA)^9^. Many MDA and RCA applications alternatively use single-stranded DNA as a starting material. After initiation, primer design in methods like LAMP enable rapid exponential amplification as the primers form loop structures to create 3′ ends extendable by a DNA polymerase. Generally, all of these methods target very short amplicons, with length usually less than 150 base pairs (bp). LAMP and rolling-circle methods (RCA, MDA) produce much longer, complex products which are concatameric, highly branched, and difficult to use in any downstream application. A limitation of isothermal methods has been the creation of discrete (i.e. a single DNA species), long (>500 bp) dsDNA products. Here we present a novel isothermal amplification scheme capable of producing discrete, several kilobase (kb)-long dsDNA products using only two specific primers, a strand-displacing DNA polymerase, and the single-stranded DNA binding protein gp32 from bacteriophage T4.

Gp32 is an essential protein that binds to single-stranded DNA (ssDNA) during phage DNA replication, recombination and repair processes. It binds ssDNA cooperatively as a monomer, and each gp32 protein covers ~7 bases of ssDNA with no observed sequence specificity^10,11^. This binding serves to maintain a transient ssDNA state during DNA replication, and facilitate the interaction with other replication/recombination proteins such as UvsX and UvsY^12–14^. The ssDNA binding activities of gp32 is essential for a number of *in vitro* assays and applications and *in vivo* functions. Gp32 is required for UvsX catalyzed strand exchange of double-stranded linear DNA with single-stranded circular DNA^15,16^. General ssDNA binding is not sufficient for these activities, as *E. coli* SSB does not support the strand exchange activity of UvsX^15^. In DNA amplification applications, gp32 has been shown to: increase PCR amplicon length^17^; improve PCR yield and DNA sequencing read length^18^; alleviate PCR inhibition^19^; and increase the yield of full-length cDNA in reverse transcription reactions^20–22^. Notably, gp32 is used in the strand-invasion based isothermal amplification RPA^6^ and SIBA^5^ protocols where it works in concert with UvsX and UvsY to invade and anneal primers into dsDNA targets enabling exponential DNA amplification.

In the above applications the effect of gp32 was mostly attributed to its ability to bind single-stranded nucleic acid, such as the oligonucleotide primers or target DNA or RNA. In this report we demonstrate that gp32 alone is able to assist strand invasion of primers into dsDNA at moderate temperatures (30–45 °C) and facilitate formation of an initiation site for DNA amplification. Using a combination of gp32 and a mesophilic strand-displacing DNA polymerase (*Bsu* DNA Polymerase, Large Fragment) we were able to amplify discrete dsDNA amplicons of several kilobases from circular templates using a pair of target-specific primers, as well as higher molecular weight products consisting of multiple repeated copies of the amplicon and template. We also demonstrate a significant increase in specificity of amplification facilitated by gp32, with a suppression of nonspecific product in the presence of the single-stranded binding protein. Together these results show an unexpected role for gp32 in facilitating strand invasion and initiation of synthesis, with a novel isothermal amplification application uniquely capable of producing several-kilobase, discrete products from circular dsDNA.

## Results

### T4 gp32 facilitates isothermal amplification

**Figure 1:**
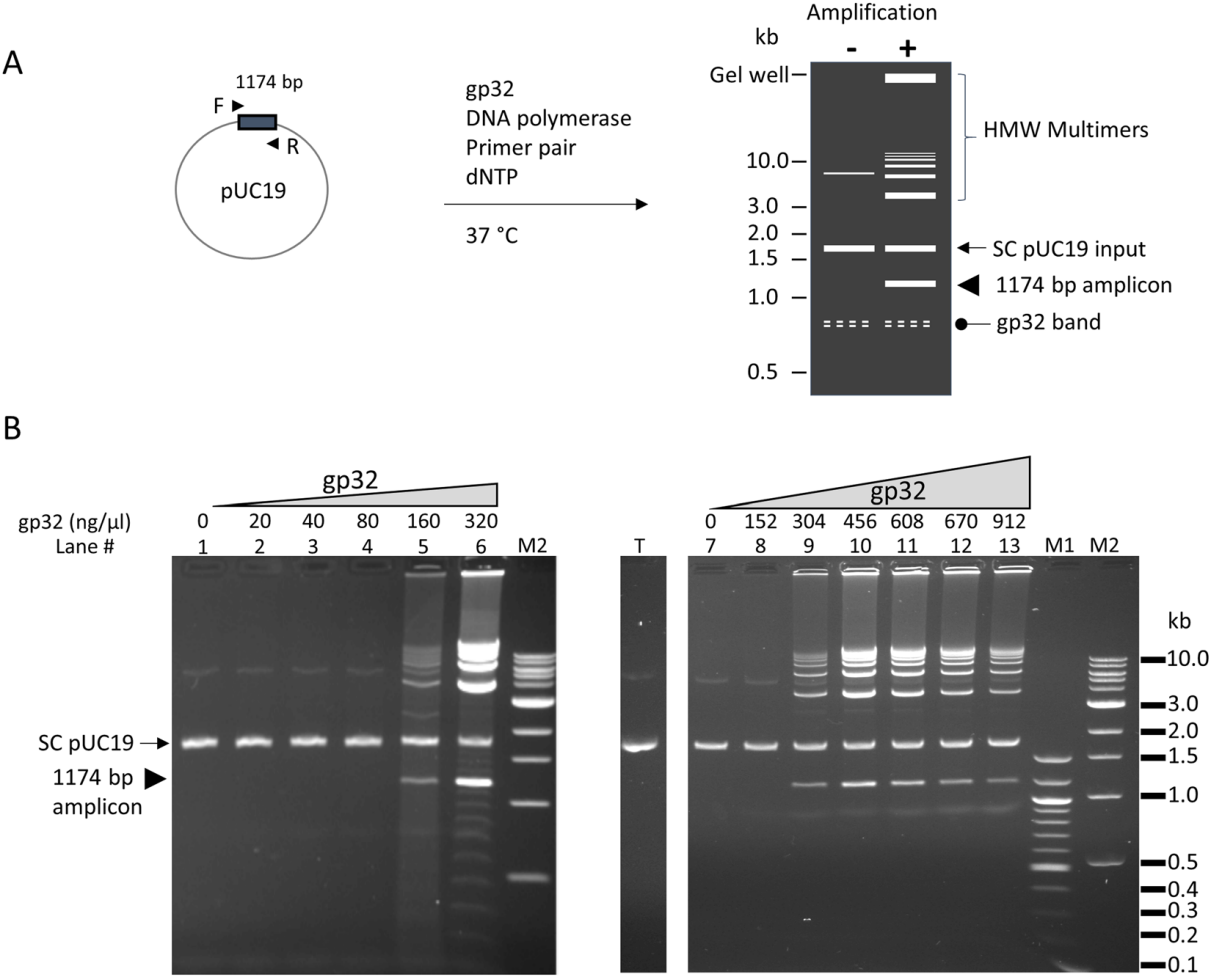
T4 gp32 protein-assisted isothermal amplification on circular dsDNA template pUC19. (**A**) schematic representation of the amplification reaction and product pattern after agarose gel electrophoresis. Primer pair consisting of the F and R primers (arrow heads) allows the amplification of a 1174 bp amplicon (box) of the pUC19 plasmid (circle). When there is no amplification, pUC19 migrates as a supercoiled band (SC pUC19, arrow) and weaker upper band. There is usually a faint and often diffuse band migrating at approximately 0.8 kb (oval arrow) whenever gp32 protein is present in the reaction. The amplification products consist of a band of the single amplicon (arrow head), high molecular weight (HMW) multimers (bracket) including those forming a ladder pattern and those retained in the gel well. HMW multimers migrate slower than the SC pUC19 input (arrow) and the linearized pUC19 (not shown). (**B**) Effect of gp32 concentration on the amplification of 0.1 μg of pUC19 template using primer #21: Left panel (lanes 1–6), low range of gp32 (20–320 ng/μl); Right panel (lanes 7–13), high range of gp32 (152–912 ng/μl). Lanes 1 and 7 contained no gp32 as negative controls. The location of the single amplicon (1174 bp) flanked by primer pair #21 is marked by an arrow head in the left panel. Lane T has 0.1 μg pUC19 alone without any treatment. M1 and M2 are 100 bp ladder and 1 kb ladder DNA markers respectively.

To assess the role of gp32 in facilitating initiation, we tested it in DNA amplification reactions containing a pair of specific primers flanking an amplicon (as designed for PCR) using circular double-stranded pUC19 plasmid template, DNA polymerase, dNTPs in a reaction buffer suitable for amplification (Fig. 1A). No thermal denaturation nor annealing steps were included, and the reactions were incubated at 37 °C, thus DNA amplification was possible only when the primers were able to create priming events that were accessible to the DNA polymerase. Discrete bands were produced only when gp32 was included in the reaction at sufficient concentration (with an optimum around 300–456 ng/μl; Fig. 1B). In all reactions containing similar concentrations of gp32, we observed a faint band at ~0.8 kb that was unchanged with template of primer addition and was not affected by amplification. Analyzing the products from the gel, the smallest bands had the size of the single amplicon flanked by the primer pairs (1174 bp), and other bands of higher molecular weight approximately corresponded to multimers of linearized pUC19. Large amounts of amplified DNA were retained in the gel well during electrophoresis, and these DNA molecules likely had a high molecular weight that prevented their migration into the agarose gel (>25 kb). It is worthwhile to note that the amount and the migration pattern of the input circular dsDNA was unaffected by the amplification reaction. Using the same conditions, single amplicons with sizes from 277 bp to 2.6 kb were efficiently amplified and generated a similar ladder of multimers (Supplemental Figs. 1–2). Primers of varying in length from 15 to 60 bases were tested and all allowed amplification with similar efficiency (data not shown).

**Figure 2:**
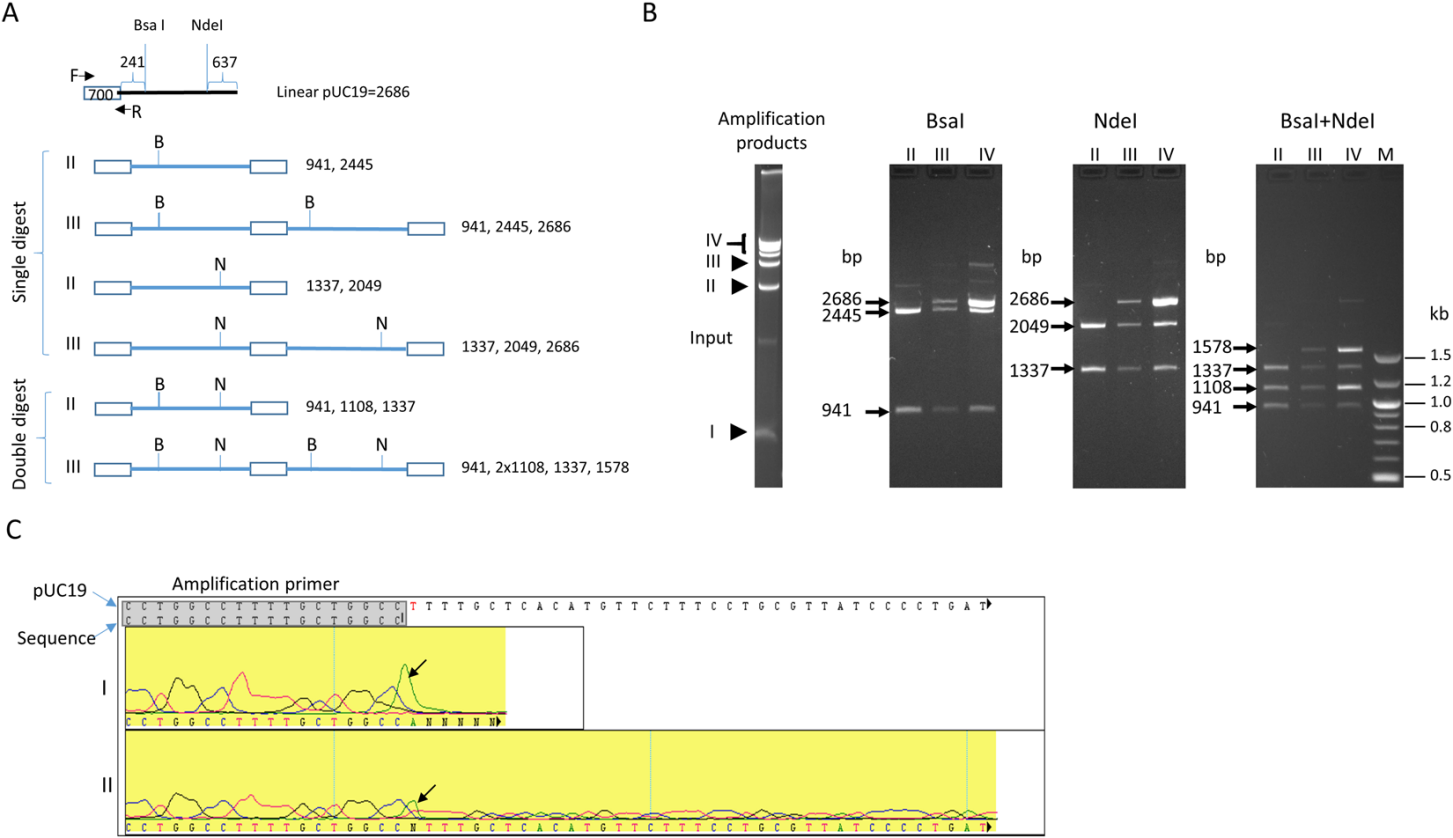
Identification of the product bands generated by gp32-mediated isothermal amplification. A primer pair (#10) generating a 700 bp fragment was used for amplification and the product was separated by agarose gel electrophoresis. (**A**) Predicted structures of amplification bands and fragment sizes with BsaI and NdeI digestion. Sizes are in bp. B, BsaI; N, NdeI. (**B**) Restriction enzyme analysis. DNA from individual amplification bands (marked as I, II, III and IV) was purified and subjected to restriction digestion with BsaI or NdeI separately or together. The digested DNA was analyzed by electrophoresis and the predicted size was marked. (**C**) Confirmation of sequence identity in amplification products. Sequence chromatogram shown is the end region obtained from bands I and II. The entire sequence of band I was covered, with continuation into template observed from the longer band II (bottom). The arrows indicate a non-templated addition of a deoxyadenosine at the end of the amplicon during sequencing.

To confirm the identity of the product species, the structures of the gp32-assisted amplification product were analyzed by restriction enzyme digestion and sequencing. Individual bands from an isothermal reaction producing a 700 bp amplicon of pUC19 were excised and purified following electrophoresis separation. The multimeric DNA products were digested with two restriction enzymes, each cutting pUC19 once, in single or double digests (Fig. 2A). The fragment sizes obtained from these digestions (Fig. 2B) confirmed that these larger products were linear DNA that contained one or multiple linearized pUC19 plasmid sequences with a duplication of the single amplicon at the opposite end. Sequencing of both the single amplicon and the larger products confirmed the composition of the short amplicon as well as the higher molecular weight bands to be the expected pUC19 region, terminating with the primer sequences (Fig. 2C).

**Figure 3:**
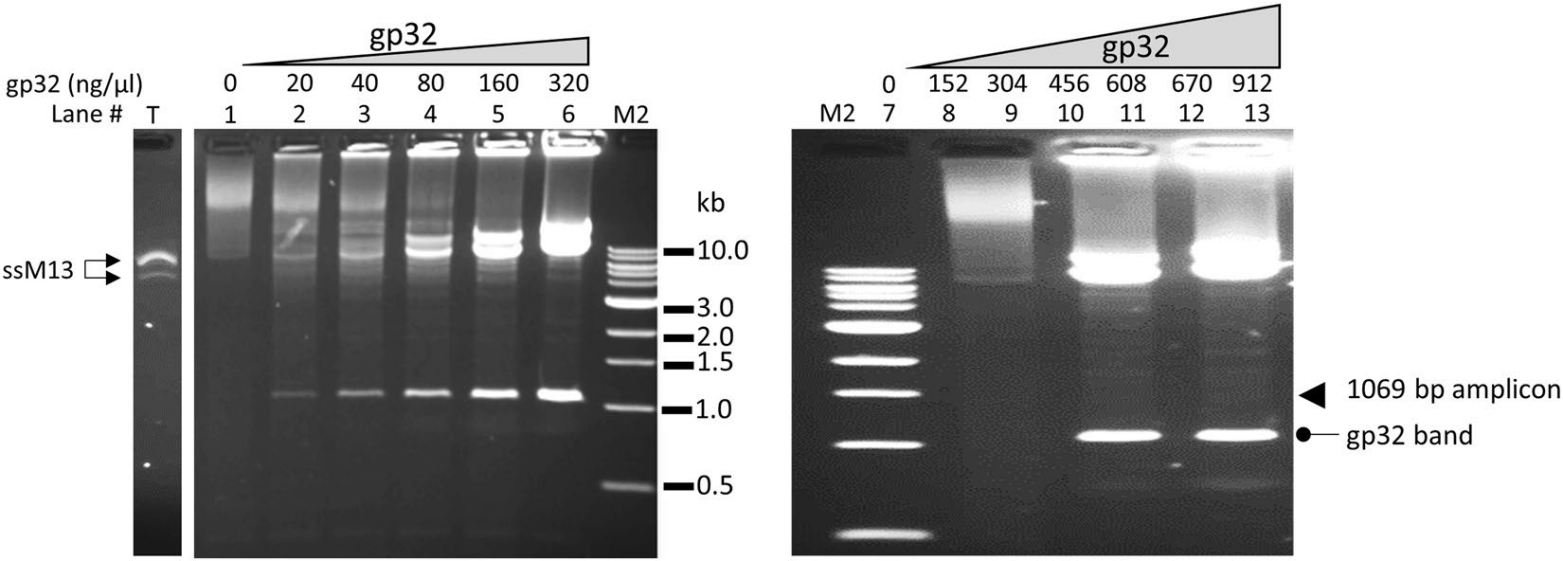
T4 gp32 protein-assisted isothermal amplification on circular ssDNA M13. Lane T was loaded with 0.1 μg ss M13 DNA alone without any treatment. The amplification of 0.1 μg of M13 ssDNA using primer # 25 with low range of gp32 (20–320 ng/μl) (left panel, lanes 2–6), or high range of gp32 (152–912 ng/μl) (right panel, lanes 8–13). Each panel has a lane with no gp32 (Lanes 1 and 7) as negative controls. The band of the single amplicon (1069 bp) is marked by an arrow in the right panel. M2 is 1 kb ladder DNA markers.

We also tested gp32-mediated amplification using single-stranded circular DNA (M13) as the template (Fig. 3). Similar amplification products as from the dsDNA template, including discrete bands corresponding to the predicted size of the amplicon, higher MW multimers and those retained in the gel loading well, were observed. A similar concentration of gp32 (~300 ng/μl) was required for highest yield, although lower concentrations tended to generate more product than that with dsDNA template. Higher concentrations of gp32 beyond this optimum reduced the product yield of all the bands proportionally as with the dsDNA template. In the absence of gp32, a smear of high molecular weight products was produced, and this smear was eliminated by the addition of increasing amounts of gp32. In contrast to the amplification with circular dsDNA where the migration or amount of input templates was not affected (Fig. 1B), the ssM13 template band disappeared from its normal location (Fig. 3) as M13 substrates were likely converted to partial or full duplexes with the newly synthesized DNA that has a slow migration. As with circular dsDNA, amplicons varying in sizes from 1.0 to 2.5 kb were amplified with similar efficiency, and even the entire 7.3 kb M13 was successfully amplified with primers designed accordingly (Supplemental Fig. 1), indicating that sizes up to 7 kb are not limiting for the amplification.

### Characterizing gp32-dependent amplification

We tested whether linear dsDNA could serve as a template. Using the same primers, we PCR-amplified the pUC19 fragment that supported gp32-based amplification (Fig. 1), and attempted the isothermal reaction using the purified PCR product as template. Using the same conditions and primers as the successful reaction with circular pUC19, no amplification was detected using the linear template. In another test, using 6 different primer pairs targeting two separate regions of phage lambda DNA did not give rise to any amplification products using linear lambda DNA templates (data not shown).

The accumulation of amplification product over time was analyzed in reactions containing either a primer pair or a single primer. With pair of primers, visible product of the single amplicon and the multimers appeared after 20 min incubation, and these products accumulated at approximately the same rate (Supplemental Fig. 2). In reactions with only a single primer, we observed high molecular weight products appearing around the same time (~20 min) of incubation, and they accumulated over time. As there is only a single specific primer in the reaction, these products are likely single stranded DNA produced from rolling-circle amplification.

**Figure 4:**
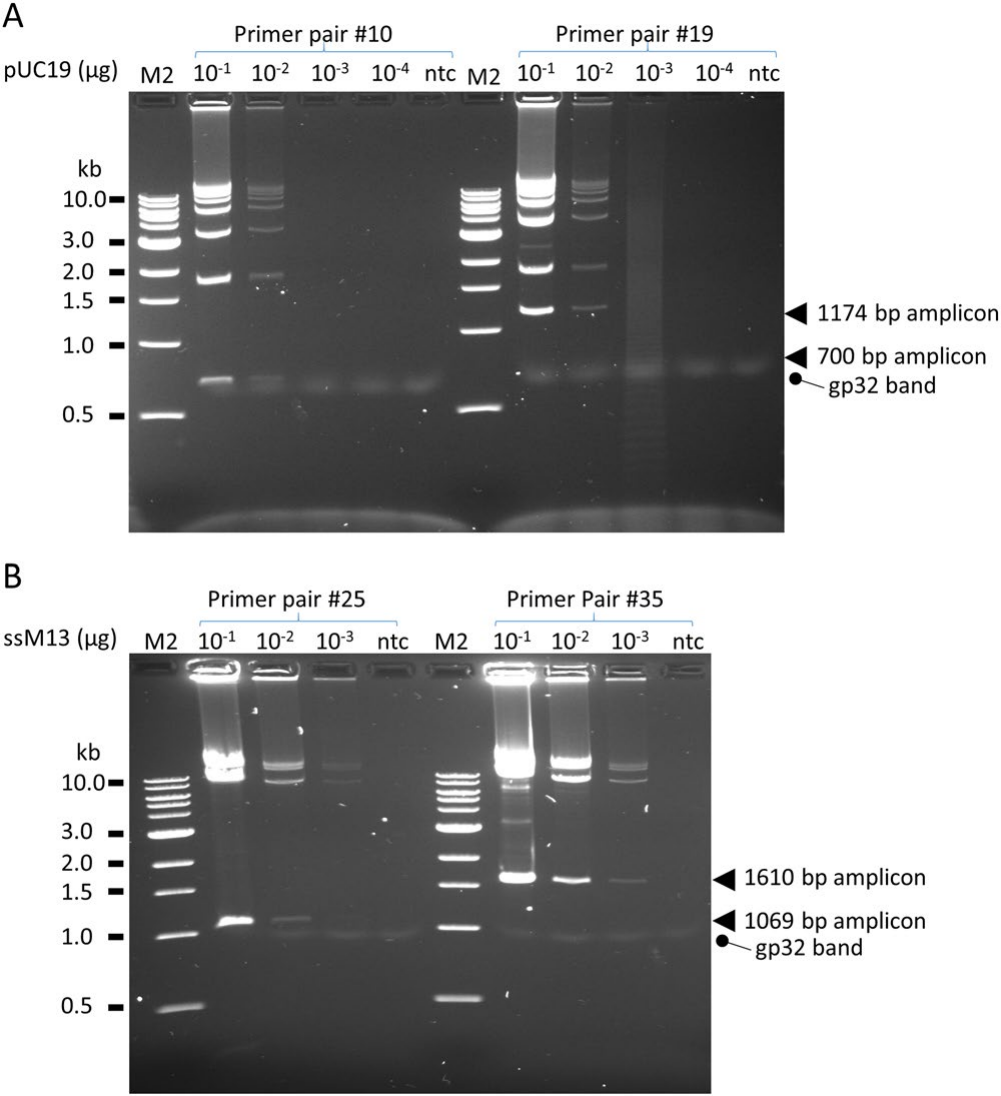
Sensitivity of gp32-assisted isothermal amplification. A. pUC19 dsDNA template with primer pair #10 or #19 which specify, respectively, single amplicons of 700 bp and 1174 bp in size. The amplification reaction contained 456 ng/μl of gp32. B. ssM13 template with primer pair #25 or #35 which specify, respectively, single amplicons of 1069 bp and 1610 bp in size. The amplification reaction contained 302 ng/μl of gp32. Arrows indicate the locations of single amplicons. Incubation time for both template was 1 hour. M2, 1 kb ladder.

The sensitivity of the amplification was determined with double- and single-stranded DNA templates (Fig. 4). Products on agarose gel were generated from 0.01 μg dsDNA template (Fig. 4A) and from ~10-fold lower input for ssDNA template (Fig. 4B). Longer incubation time proportionally increased the yield but the rate of product accumulation does not appear to be exponential (data not shown).

We examined different polymerases for compatibility with the reaction, and incubated the reactions at a range of temperatures according to their preferences. We found that several strand displacing DNA polymerases were able to support the amplification. For example, Klenow (*E. coli* DNA polymerase I, large fragment) produced the same amplification patterns as *Bsu* and the maximum activity was observed at approximately 38 °C (Supplemental Fig. 3), which is optimal for both gp32 and Klenow. The more thermophilic *Bst* 2.0 DNA polymerase^23^ also generated the same amplification pattern but yielded the most products at temperatures of 45–48 °C. This temperature is lower than the optimal temperature of *Bst* 2.0 (60–65 °C), and this result indicates *Bst* 2.0 retains enough polymerase activity at the lower, optimal temperature for gp32. Two strand displacing polymerases, phi29 DNA polymerase, commonly used in traditional rolling-circle amplification, and Vent (exo-) DNA polymerase were unable to support gp32-mediated amplification (Supplemental Table 3). Additionally, two other non-strand displacing polymerases, *E. coli* DNA polymerase I and T4 DNA polymerase (Supplemental Table 3) did not support gp32-mediated isothermal amplification. Thus, strand-displacement activity is required for the amplification, but is not sufficient, as phi29 and Vent (exo-) failed to produce any of the discrete or multimeric bands.

In the interest of confirming that gp32 was indeed facilitating primer invasion into double-stranded DNA, a number of experiments were conducted to confirm that the amplification reaction was occurring from true double-stranded, closed-circular plasmid DNA templates. Several treatments were further performed to remove any nicked or any gapped forms: T4 DNA ligase to seal potential nicks, PreCR DNA repair mix to fill in any gaps and seal nicks (also repairing potential base damage), and T5 exonuclease to remove plasmid that has nicked or gapped sites. None of these treatments affected the ability of plasmid to serve as a template for efficient amplification (Supplemental Fig. 4), supporting that the double-stranded closed circles served as the template for amplification.

As single-strand breaks (i.e. nicks) on dsDNA could serve as efficient initiation sites for the strand-displacing DNA polymerase, we investigated the effect of gp32 with nicked circular dsDNA, and whether nicking can facilitate the gp32-assisted amplification (Supplemental Fig. 5). Without gp32, nicked circular DNA yielded product of various sizes was independent of the presence of primers as expected based on nick initiation. When gp32 was included, primer pairs generated significantly more product as compared to no-gp32 reactions with the appearance of specific bands corresponding to the expected amplicon products. With nicked DNA, more abundant specific bands as compared to reactions with supercoiled template were made. Single primers had a product pattern different from that without gp32, likely due to increased primer binding specificity discussed further below.

### Specific priming in gp32-dependent amplification

**Figure 5:**
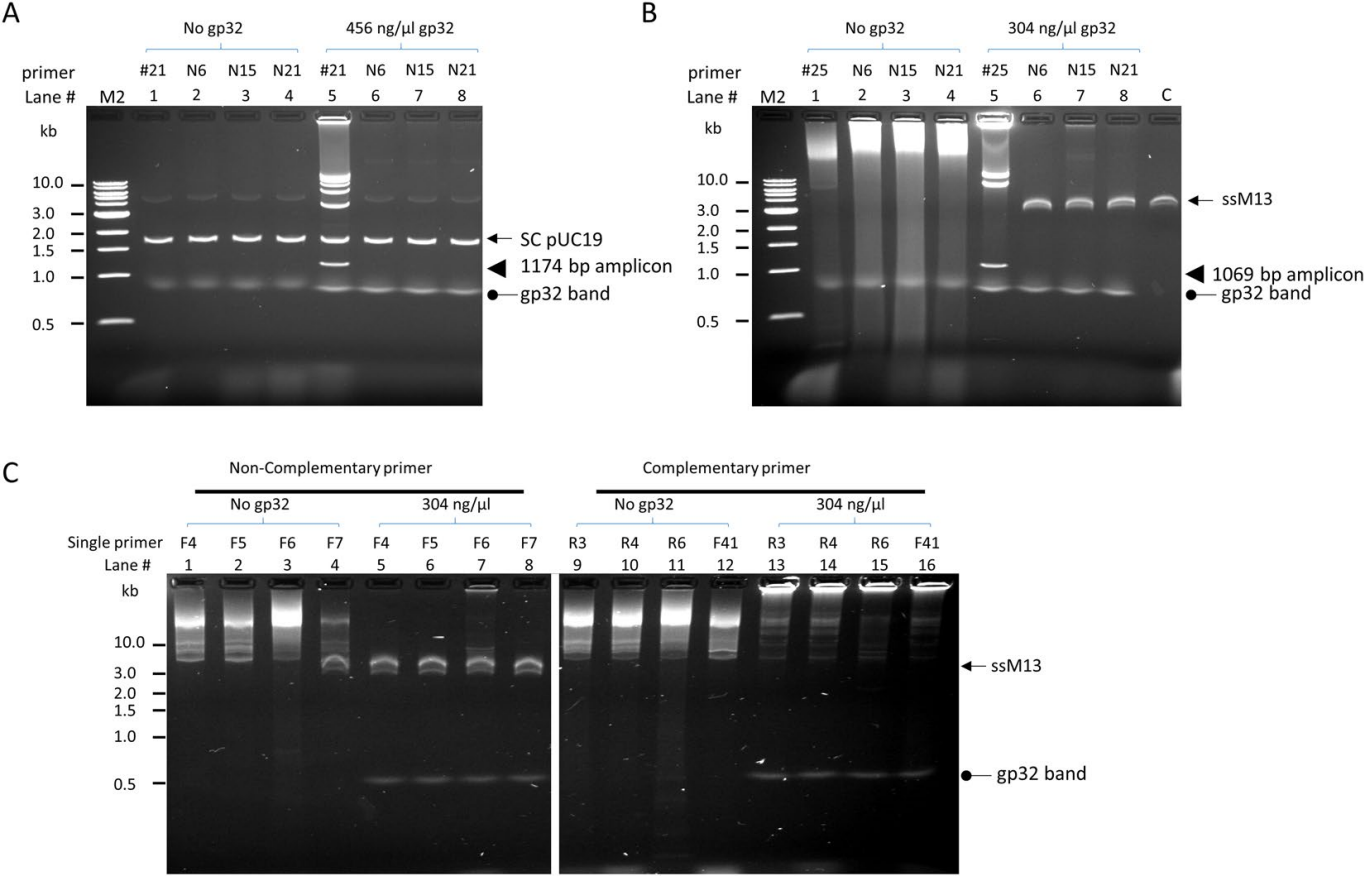
Probing the role of gp32 in conferring amplification specificity. Specific or non-specific primers were used to perform typical amplification reactions with 0.1 µg pUC19 or ssM13 DNA. (**A**) pUC19 dsDNA template with specific and random primers. Primer pair #21 produced specific product only in the presence of gp32 (lane 1 vs 5). Random primers of various length (N6, N15 or N21) gave no product regardless of the presence of gp32 (lane 2–4 and 6–8). (**B**). M13 ssDNA template with specific and random primers. Without gp32, high molecular weight smear products were generated with either a primer pair or random primers (lanes 1–4). With gp32, primer pair #25 (lane 5) gave typical specific bands while random primers of various lengths yield no amplification product (lane 6–8). Lane C, untreated ssM13 DNA. (**C**) Amplification reactions with single primers that have either the same (lanes 1–8) or complementary sequences to ssM13 (lanes 9–16). In the absence of gp32 (lanes 1–4 and 9–12), primers having either the same sequence or complementary sequences produced high molecular weight amplification products. Including gp32 in the reaction prevented nonspecific amplification with non-complementary primers (lanes 5–8) and allowed the generation of even larger product with complementary primers (lanes 13–16).

A difference between the gp32-mediated reaction and traditional rolling-circle reactions is the use of PCR-like gene-specific primers in place of random hexamers as commonly used in RCA. To demonstrate the role of primer specificity, we compared the performance of random primers to the specific primers. Without gp32 protein, neither specific primer pairs nor random primers of various length (from 6- to 21-mers) supported any amplification from ds pUC19 (Fig. 5A, lanes 1–4). When gp32 was included, specific primer pairs gave the expected bands (Fig. 5A, lane 5), while random primers still produced no visible product (Fig. 5A, lanes 6–8). Interestingly, with single-stranded M13 template in the absence of gp32, both specific and random primers produced a smear of high molecular weight products (Fig. 5B, lanes 1–4) and the input ssM13 band disappeared from its original location. These smear products required ssDNA template as they were not present when dsDNA was present (Fig. 5A) or when there was no template DNA (data not shown). In the presence of gp32, the smear produced by both random primers and primer pairs was abolished (Fig. 5B, lanes 5–8) and left the input ssM13 intact, and the primer pair generated only the anticipated specific bands (Fig. 5B, lane 5). These results demonstrate that gp32 enhances binding of specific primers with circular ssDNA and thus allows the production of bands specified by a primer pair.

This enhanced specific binding by gp32 was further examined using single primers that either had the same sequence as the ssM13 template (with no specific annealing to the template) or were complementary to it (with a single specific annealing site in the template). In the absence of gp32, these two types of single primers produced a similar smear of high molecular weight product (Fig. 5C, lanes 1–4 for non-complementary primers and lanes 9–12 for complementary primers) and the smear is similar to that produced with random primers without gp32 (Fig. 5B, lanes 2–4). Similar results from both primer types indicates a high level of nonspecific amplification in the reaction. In the presence of gp32, these two types of primers produced very different amplification results. No amplification was observed with single primer that had the same sequence as the template (Fig. 5C, lanes 5–8) and left the ssM13 input intact, indicating gp32 prevented the nonspecific priming and amplification via the non-complementary oligonucleotide. In contrast, the single complementary primers produced high molecular weight products of which most remained in the wells of the agarose gel, while the input ssM13 band disappeared in these reactions (Fig. 5C, lanes 13–16). The migration pattern of these high molecular weight products was noticeably different from the smear produced without gp32 (Fig. 5C lanes 9–12). It is likely that these products were initiated at the single annealing site by the complementary primer and synthesized as long ssDNA that also formed a partial duplex with the input ssM13, and resulted in slow migrating molecules. In contrast, without gp32 it made no difference for primers with or without complementary sites and both could form transient priming at multiple sites and initiate DNA synthesis. These results further indicate that gp32 ensures homologous pairing between a complementary primer and its single stranded template and prevents non-specific annealing.

## Discussion

**Figure 6:**
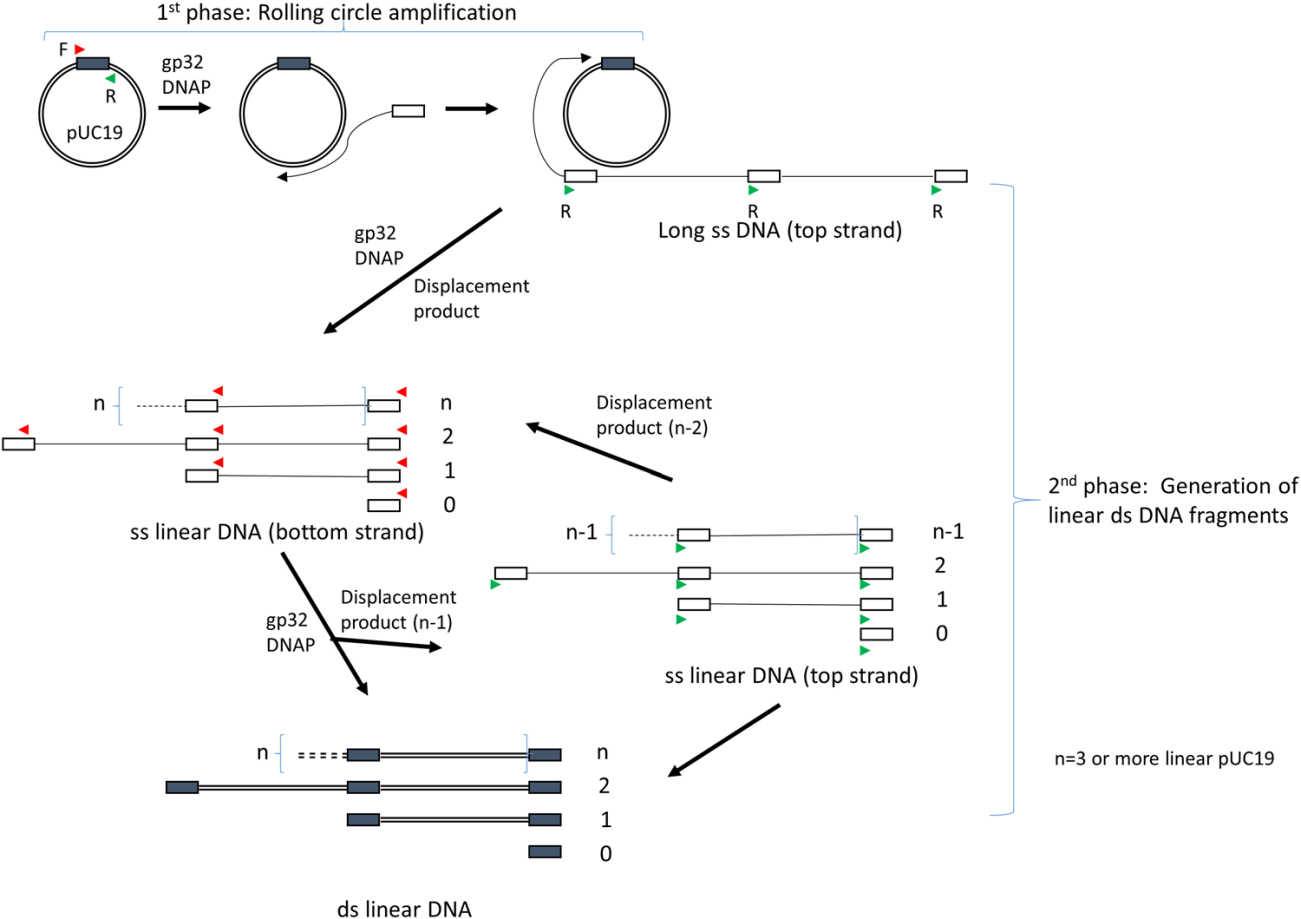
A model of gp32-assisted isothermal amplification on circular templates. The amplicon (depicted as a box) is specified by a pair of primers (forward, F, red arrow and reverse, R, green arrow) on the circular ds pUC19 template (circle). The amplification is proposed to occur in two phases. In the first phase, with the help of gp32 protein both primers (for simplicity only the product from F primer is shown) invades dsDNA and initiates DNA synthesis by a strand-displacing DNA polymerase to produce long single-stranded DNA corresponding to the top strand sequence of pUC19. For circular ssDNA template, this phase occurs similarly but does not require a strand invasion step. In the second phase, which is also facilitated by gp32, this single stranded product serves as the template for the reverse primer (R, green arrow) for DNA synthesis in the opposite direction. As there is one primer site at each amplicon for every length of the pUC19, discrete sizes of ssDNA products (bottom strand) are generated through strand displacement synthesis at multiple sites and they consist of a single amplicon and larger species of one or more entire length of the linear templates (n) linked with a single amplicon at one end. After another round of priming and DNA synthesis by the other primer (red arrows), these ssDNA species are then converted to dsDNA fragments which migrate as discrete bands in agarose gels. At the same time, ssDNA species of the opposite strand (top strand) are also generated in a similar displacement synthesis but one linear pUC19 shorter (n-1) and they are converted to ds fragments in subsequent round of synthesis.

Here we show that in the presence of gp32 protein, a pair of sequence-specific primers can be used for amplification of dsDNA templates at 37 °C without denaturation or supplemental recombination enzymes. The experiments described above suggested a model of how amplification occurs with gp32 and a DNA polymerase on circular dsDNA without denaturation (Fig. 6). The amplification happens in two phases: first, facilitated by gp32, primers invade into dsDNA and establish priming sites stable enough for the DNA polymerase to initiate DNA synthesis. Continued DNA synthesis is achieved by the strand-displacing DNA polymerase and long single-stranded products form in a mechanism similar to rolling circle amplification. In the second phase, the newly synthesized ssDNA serves as the template for the reverse primer to anneal and start DNA synthesis in the opposite direction. The reverse primer annealing site is available at the end of the amplicon between each length of the pUC19. This cycle of amplification is supported by the generation of single-stranded products in the presence of single primer at approximately the same time as initial appearance of the discrete amplicons (Supplemental Fig. 2). Further priming and DNA synthesis converts these ssDNA strands into fragments of dsDNA with discrete sizes, with each carrying a forward and a reverse primer at their opposite ends. In reactions using circular ssDNA template, the amplification proceeds similarly through these two steps except that the rolling circle amplification phase occurs with ssDNA template which is likely more accessible for primer annealing. Nevertheless, gp32 is also required for specificity and efficient amplification with ssDNA templates.

An important aspect of this amplification model explains the requirement for circular DNA. The initiation and amplification should works just as well using linear DNA input, but as we described above attempts to use linear input were unsuccessful. The initial ssDNA produced by rolling circle is concatermeric so there are multiple sites for the reverse primer to bind and initiate replication. When synthesis from upstream primers reaches a downstream primer, this downstream strand will be displaced as ssDNA and available for next round priming and DNA synthesis. In contrast, with linear dsDNA template, this is not possible and all DNA synthesis depends solely on gp32-assisted melting and priming on dsDNA. We imagine this initiation as the rate-limiting step and, accordingly relying on the slow initiation for every cycle of amplification results in the failure to generate a detectable amplification product with linear DNA. Supporting this model, nicked circular DNA proved an even better substrate for amplification, resulting in production of discrete DNA amplicons more quickly and with higher yield. We attribute this result to a more-rapid initiation and synthesis by the DNA polymerase, as no primer invasion was required to begin amplification but rather the nick provided an efficient substrate without any gp32 facilitation. However, the specificity enhancement of gp32 were required to give the discrete products even with gp32, further demonstrating its dual role in enabling amplification

The most unexpected feature of this amplification is that it occurs at 37 °C where there is no thermal denaturation to allow primer binding to template. Instead, the binding of the primer is assisted by a high concentration of gp32 protein. With dsDNA, gp32 likely is required for strand invasion into dsDNA template. This strand invasion could occur by gp32-bound oligos annealing to targets by taking advantage of temporary DNA relaxation, or by gp32 protein stabilizing ssDNA in the duplex. With ssDNA target or during the second phase of amplification (Fig. 6), gp32 appears to play a role in ensuring primer specificity. This may be achieved in such a way that only properly base paired priming sites are stable enough for initiation of DNA synthesis. This action would prevent initiation of DNA synthesis from less-stable transient priming that contributes to non-specific amplification as with random primers.

The amplification we observed is similar to, but fundamentally different from, the well-known rolling circle amplification (RCA). The most common form of this technique, multiply-primed RCA, utilizes random hexamers annealing to denatured double stranded plasmid at multiple sites that enables amplification by phi29 DNA polymerase^9^. The amplification products are a mix of double- and single-stranded DNA with no defined lengths that migrate as a high molecular weight smear in agarose gels. In contrast, in our reactions with gp32, neither random hexamers nor phi29 produced any product with double-stranded plasmid template. Another form of RCA, called hyper-branched RCA^24^, has been demonstrated using short circularized ssDNA called padlock circles as template with a pair of primers using Vent (exo-) DNA Polymerase and an incubation temperature of 60–65 °C. This method generates a ladder which was interpreted as simple multimers of linearized circle^24^. These reactions also included gp32 in the reaction, although at 5–10 fold lower concentrations than used in experiments detailed here. When this hyper-branched RCA was carried out using *Bst* DNA polymerase at similar temperature, no gp32 was required^25,26^. In contrast, the amplification described here works with both double- and single-stranded circular DNA templates, the reaction occurs at 37 °C and requires a high concentration of gp32 (~300 ng/μl). Analyses of the products indicate the smallest band is a discrete amplicon flanked by the opposing primer pair, and the rest of the ladder consists of concatamers of the linearized circle with a duplication of the single amplicon (Fig. 6).

Similarly high concentrations of gp32 were required in RPA (~900 ng/μl) and SIBA (~250 ng/μl)^5,6^ where two other T4 proteins, UvsX and UvsY, are also required. Both amplifications take advantage of the primer binding and dissociation cycles facilitated by UvsX to achieve exponential PCR-like amplification even with a linear template, although they adapt different strategies in primer designs. Both RPA and SIBA target relatively short amplicons. RPA prefers short amplicons (around 500 bp or shorter) for efficient amplification while SIBA only works with very short amplicons due to the requirement for a synthetic oligonucleotide for strand invasion. As we demonstrated, we were able to amplify amplicons of several kb in length in a simple system without the need for recombination enzymes, invasion oligo templates, or associated ATP regeneration systems. Products of this size and nature are much more easily utilized in downstream applications (e.g. cloning, sequencing) and isothermal production of these products could enable clonal amplification of longer library inserts than is currently possible in next-generation sequencing platforms. Moreover, this gp32-mediated amplification also generated very long products (>25 kb), demonstrating a combination of benefits from synthesizing discrete amplicons generated by methods like SDA and RPA and high molecular weight products generated by LAMP and RCA.

In summary, we have demonstrated that with the assistance of a high concentration of gp32, it is possible to generate discrete dsDNA products at 37 °C using only single-stranded binding protein, DNA polymerase, and two target-specific primers. This study reveals that gp32 has some unexpected roles in promoting primer invasion on a dsDNA template, enhancing specific binding and preventing non-specific primer annealing. These properties may generally facilitate DNA amplification strategies at ambient and moderate temperatures. The ability to synthesize long, discrete dsDNA at low temperature expands the reach of DNA amplification methods and may have use in molecular diagnostics, clonal amplification for next-generation sequencing (particularly where long DNA is desired, e.g. nanopore and Pacific Biosciences methods), and other biotechnology applications. Though the method requires relatively high input amount compared to sensitive diagnostic amplification techniques, the ability to produce long, discrete and concatameric products from a small and easily-prepared substrate would be useful in many of these methods. Although at this time it can only efficiently utilize circular DNA templates, future testing will determine the feasibility of extending the method to amplifying linear DNA as in PCR, but without the need for DNA denaturation by thermal cycling.

## Materials and Methods

Amplification reactions were performed in 50 mM Tris-Acetate, pH 7.9, 50 mM potassium acetate, 7 mM magnesium acetate, 2 mM DTT, 0.6 mM each dNTP, 1.0 μM each forward and reverse primer, 0.33 U/μL *Bsu* DNA Polymerase, Large Fragment (New England Biolabs (NEB)) and 300–456 ng/μl (9.0–13.6 µM) T4 gene 32 protein (NEB). DNA oligos were from IDT and their sequences and target amplicons are listed in Supplemental Materials (Supplemental Tables 1–2). DNA templates (NEB) were pUC19 plasmid DNA and M13mp18 single-stranded DNA. Reactions were incubated at 37 °C for 1 hour unless otherwise specified.

Other polymerases tested were from NEB unless otherwise noted: *Bst* 2.0; *Bst* 3.0; DNA Polymerase I, Large (Klenow) Fragment; Sequenase Version 2.0 DNA polymerase (Affymetrix); phi29 DNA Polymerase; DNA Polymerase I (*E. coli*); T4 DNA Polymerase.

Amplification reactions were stopped by addition of 1x SDS-containing loading buffer and analyzed by 1x TBE agarose (1%) gel electrophoresis containing ethidium bromide. DNA markers were 100 bp ladder or 1 kb ladder (NEB). For analyzing the structure of the amplified products, individual bands were excised from the gel and DNA was extracted using a Monarch® DNA Gel Extraction Kit (NEB). Purified DNA was then used for restriction analysis with either one or two restriction enzymes and for Sanger sequencing.

### Data Availability

No datasets were generated or analyzed under the current study.

## Electronic supplementary material

Supplemental Material
