# Author Correction: Isothermal Amplification of Long, Discrete DNA Fragments Facilitated by Single-Stranded Binding Protein

**DOI:** 10.1038/s41598-018-22186-z

**Published:** 2018-03-02

**Authors:** Yinhua Zhang, Nathan A. Tanner

**Affiliations:** New England Biolabs, 240 County Road, Ipswich, MA 01938 USA

Correction to: *Scientific Reports* 10.1038/s41598-017-09063-x, published online 17 August 2017

This Article contains an error in Figure 3, where the gel image is formatted incorrectly. The correct Figure 3 appears below as Figure 1.

**Figure 1:**
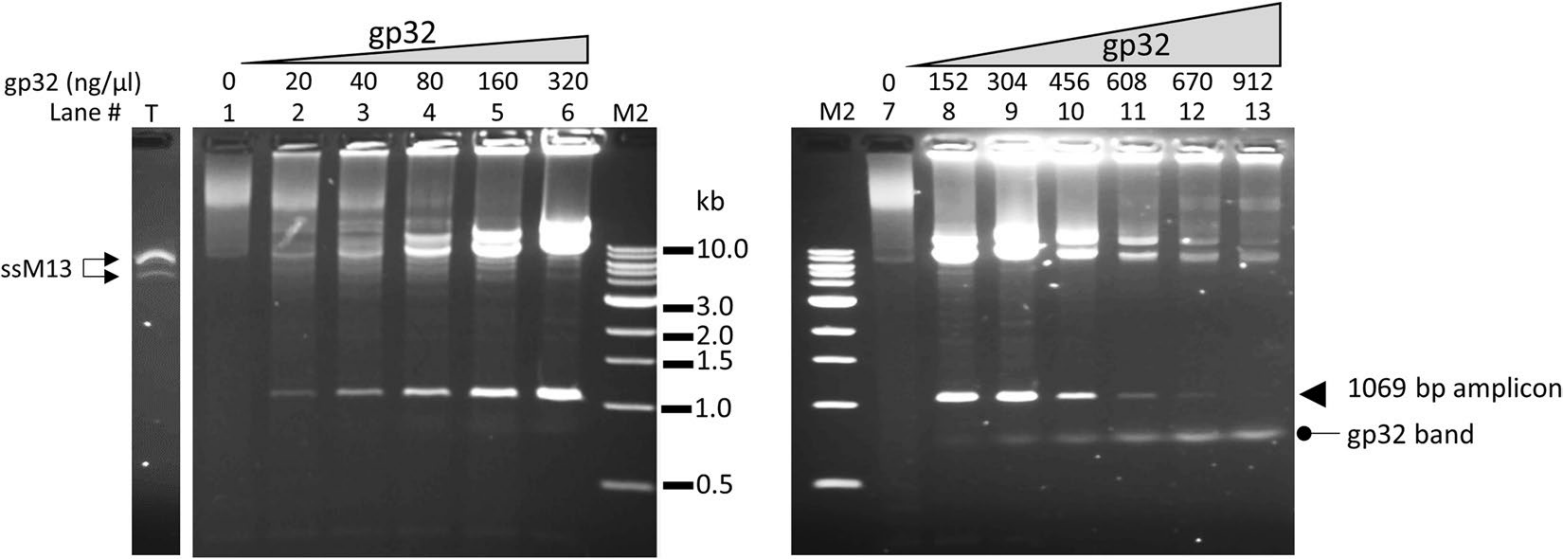
T4 gp32 protein-assisted isothermal amplification on circular ssDNA M13. Lane T was loaded with 0.1 μg ss M13 DNA alone without any treatment. The amplification of 0.1 μg of M13 ssDNA using primer # 25 with low range of gp32 (20–320 ng/μl) (left panel, lanes 2–6), or high range of gp32 (152–912 ng/μl) (right panel, lanes 8–13). Each panel has a lane with no gp32 (Lanes 1 and 7) as negative controls. The band of the single amplicon (1069 bp) is marked by an arrow in the right panel. M2 is 1 kb ladder DNA markers.

