# Supplementary material for: Isothermal Amplification of Long, Discrete DNA Fragments Facilitated by Single-Stranded Binding Protein
Source: Sci Rep. 2017 Aug 17;7:8497. doi: 10.1038/s41598-017-09063-x (PMC5561150; doi:10.1038/s41598-017-09063-x)
Supplement: Supplementary file 1 — Supplemental Material [file 41598_2017_9063_MOESM1_ESM.pdf]

Supplemental Material for:

Isothermal Amplification of Long, Discrete DNA Fragments Facilitated by Single-Stranded Binding Protein

Yinhua Zhang and Nathan A. Tanner*

New England Biolabs, 240 County Road, Ipswich MA 01938 USA

*Corresponding tanner@neb.com

Supplemental Figure 1

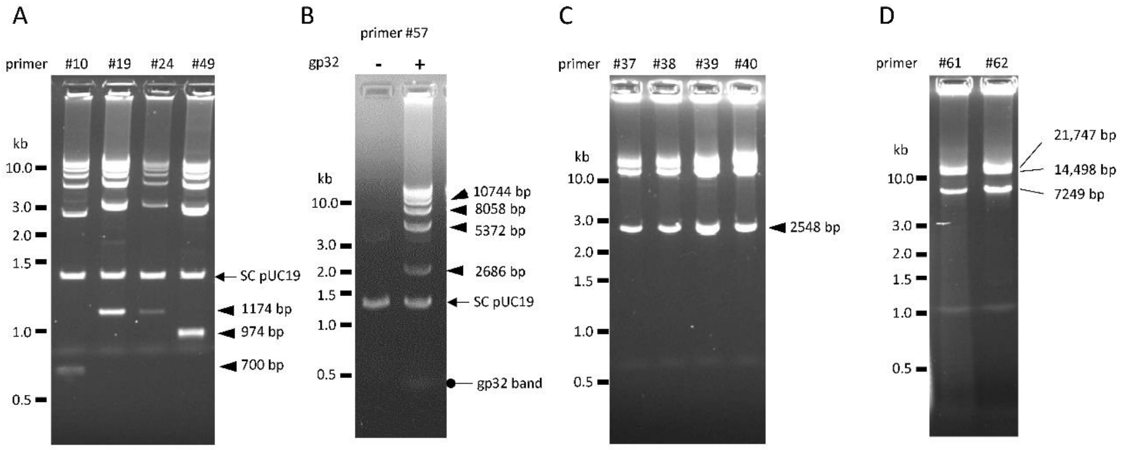

Supplemental Figure 1. Amplification of amplicons with various sizes. (**A**) 700 bp -1174 bp amplicons from pUC19. (**B**) Amplicon including of the entire length (2686 bp) of pUC19. (**C**) 2548 bp amplicon from ssM13 DNA using primers of different lengths. (**D**) Amplicons consisting of the entire length (7249 bp) of ssM13. Templates were either 0.1μg pUC19 or ssM13 and primer information is included in Supplementary Table 1-2.

Supplemental Figure 2

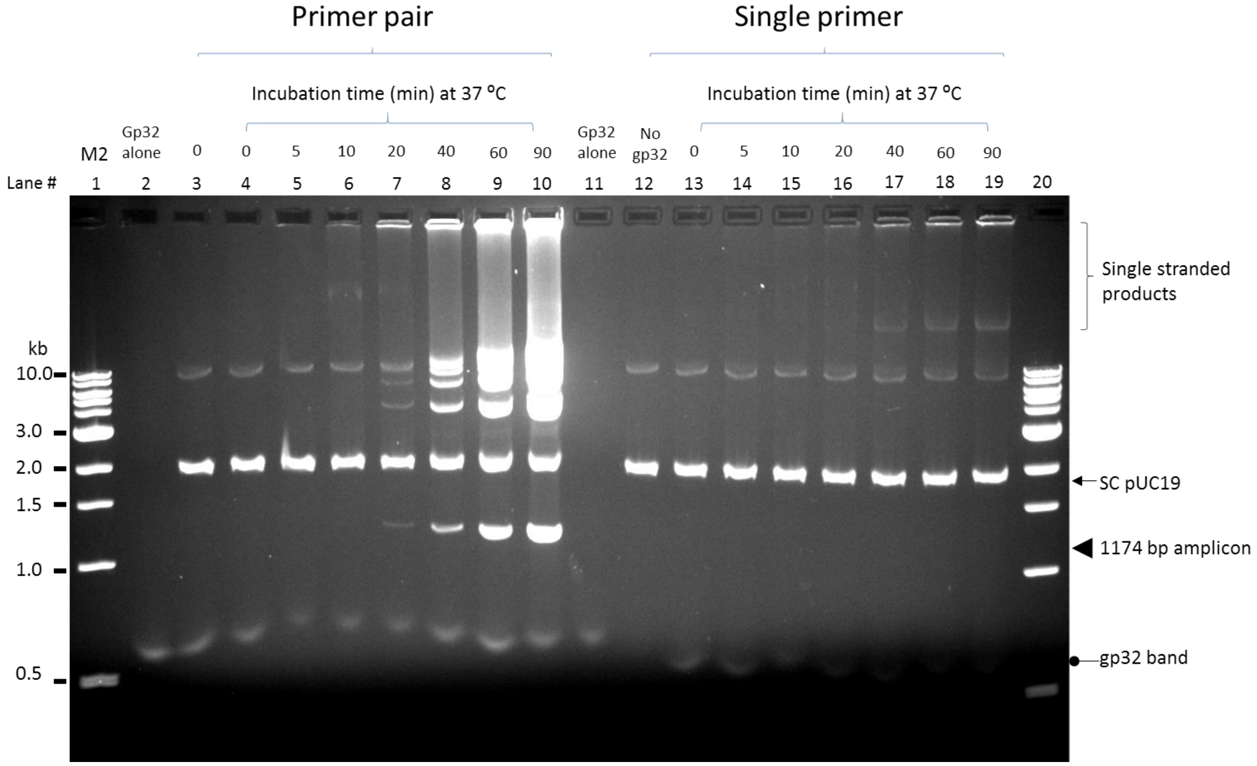

Supplemental Figure 2. Timer-course of product accumulation. A reaction mix with primer pair #21 or a single primer from this pair (F23) was set up on ice and then aliquot to PCR tubes. The reaction was then incubated at 37 ºC for different times (0, 5, 10, 20 40 60 and 90 minutes) and stopped by adding SDS-containing loading buffer. Gp32 alone (lanes 2 and 11) was loaded with just gp32 protein of the same amount. No gp32 lane (Lane 12) contained all the reaction components but gp32.

Supplemental Figure 3

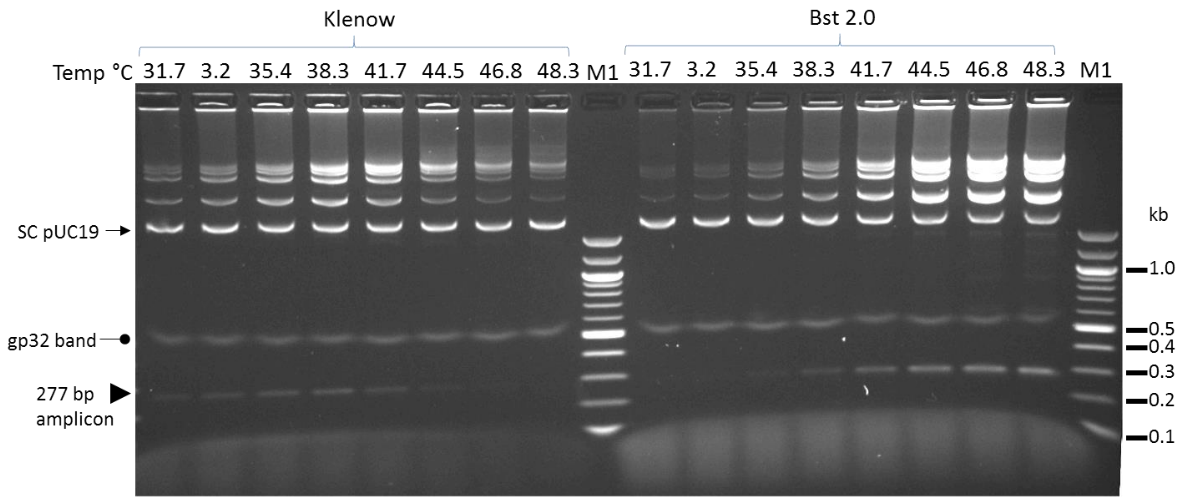

Supplemental Figure 3. Activities of DNA polymerase in gp32-assisted isothermal amplification. Examples of amplification are shown with Klenow or *Bst* 2.0 DNA polymerases. The reaction was incubated at a temperature gradient from 31.7 °C to 48.3 °C in a thermal cycler for an hour and equal amount of the product was loaded in the agarose gel. Template was 0.1μg pUC19 and primer pair #A which specifies a 277 bp fragment. M1, 100bp ladder.

Supplemental Figure 4

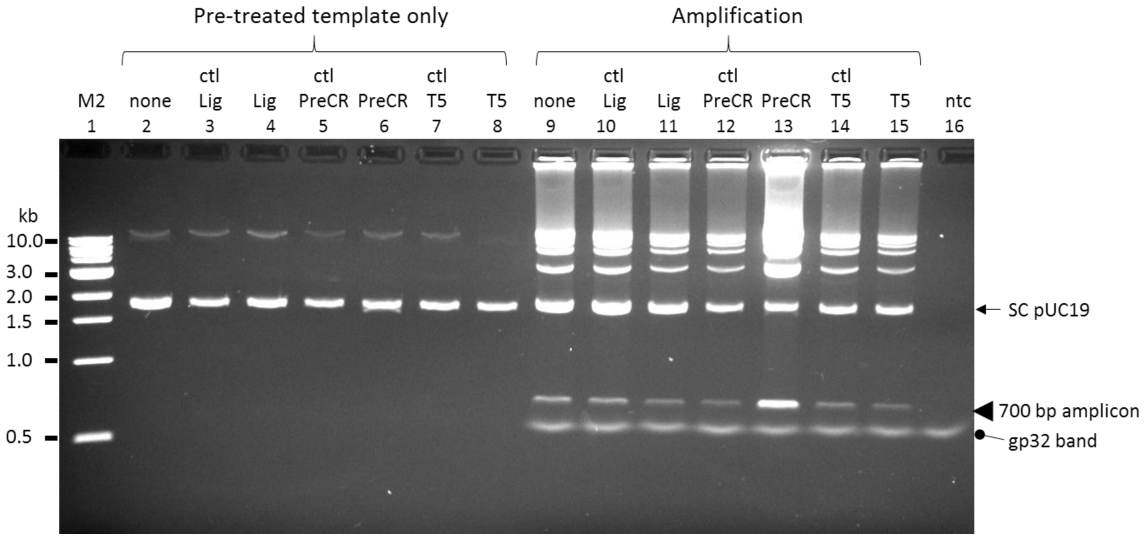

Supplemental Figure 4. Verification that amplification occurs with circular dsDNA templates. Plasmid DNA was treated with enzymes that eliminate nicked plasmid and then used for amplification. 5µg of pUC19 was treated in a 50µl reaction with respective buffer condition without or with enzyme (2 µl of T4 DNA ligase (M0202S, 400U/µl), 1ul PreCR repair mix (M0309S) or 2ul T5 exonuclease (M0306S, 10U/µl) and incubated for an hour at 37 °C. Treated plasmid was purified using Monarch® PCR & DNA Cleanup Kit and dissolved in 40 µl of TE. These treatments have no significant effect on the quantity or the migration pattern of plasmid when visualized on agarose gel (lanes 2–8). 1ul of this treated plasmid DNA (~0.1µg) was used for amplification reaction along with untreated plasmid using primer #10. No reduction of amplification with these treatments was observed (lanes 9–15), indicating circular plasmid DNA indeed serve as template for amplification. Interestingly, more amplification product was produced with plasmid treated with PreCR enzyme mix (lane 13). This suggests repairing DNA damages in plasmid allow more efficient amplification.

Supplemental Figure 5

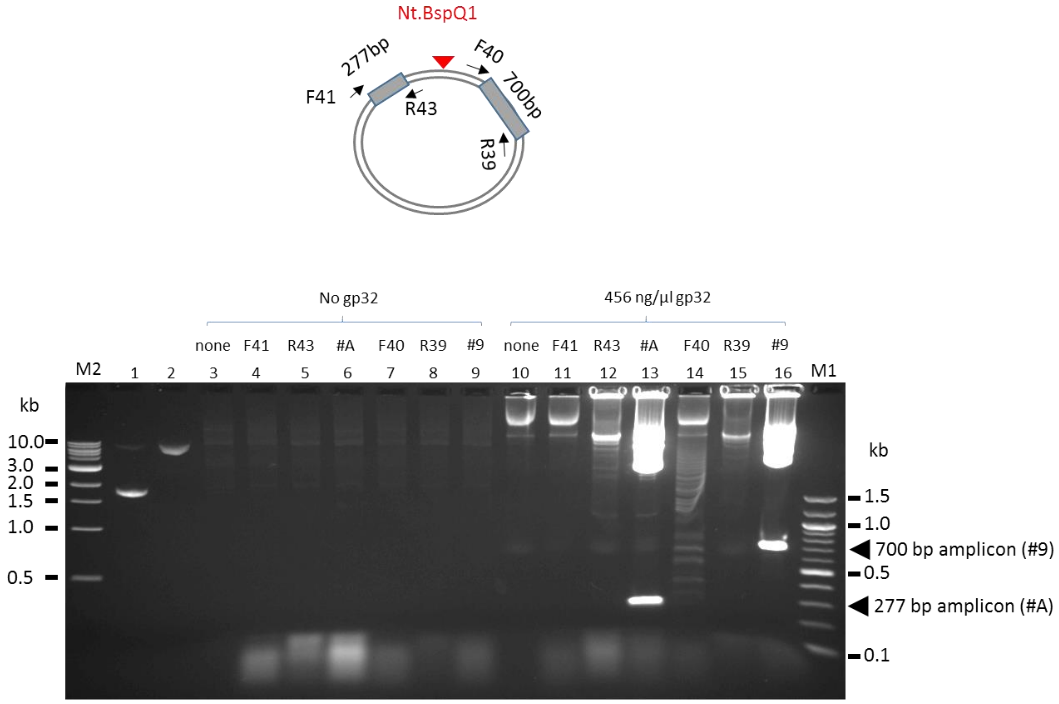

Supplemental Figure 5. Effect of gp32 on nicked circular template. pUC19 DNA was digested with nicking enzyme Nt.BspQI, which has a single site in pUC19. The two amplicons (#A and #9) are located on both side of this nicking site. After digestion, the DNA was purified and ~0.1µg used for amplification reaction without (lanes 3-9) or with gp32 (lanes 10-16) and with no primer (lanes 3 and 10), single primers (lanes 4-5, 7-8, 11-12,14-15) or primer pairs (lanes 6, 9, 13, 16). Lane 1 and 2 have ~0.1µg of supercoiled pUC19 and purified nicked pUC19 respectively.

Supplemental Table 1. Sequence of primers

Primer	Sequence	target
700R30	5'TCCCTTAACGTGAGTTTTCGTTCCACTGAG	pUC19
60-mer	5'GGGTAACGCCAGGGTTTTCCCAGTCACGACGTTGTAAAACGACGGCCAGTGAATTCGAGC	pUC19
500-41	5'CCAAATACTGTTCTTCTAGTGTAGCCGTAGTTAGGCCACCA	pUC19
F23	5'GGGTAACGCCAGGGTTTTCCCAG	pUC19
F30	5'GGCCAGCAAAAGGCCAGGAACCGTAAAAAG	pUC19
F40	5' GGCCAGCAAAAGGCCAGGAACCGTAAAAAGGCCGCGTTGC	pUC19
F41	5'GGGTAACGCCAGGGTTTTCCCAGTCACGACGTTGTAAAACG	pUC19/M13
R3	5'AACTTAATCGCCTTGCAGCACATCCCCC	pUC19
R39	5'TCCCTTAACGTGAGTTTTCGTTCCACTGAGCGTCAGACC	pUC19
R-43-mer	5'ACAGGTTTCCCGACTGGAAAGCGGGCAGTGAGCGCAACGC	pUC19
F1-22	5'CACTATTGACTCTTCTCAGCGT	M13
F4-23	5' GGTTTCTACATGCTCGTAAATTA	M13
F5-27	5'GGTTTCTACATGCTCGTAAATTAGGAT	M13
F6-32	5'GGTTTCTACATGCTCGTAAATTAGGATGGGAT	M13
F7-15	5'GGTTTCTACATGCTC	M13
R1-25	5'ACATTGGCAGATTCACCAGTCACAC	M13
R2-31	5'ACATTGGCAGATTCACCAGTCACACGACCAG	M13
R3-22	5'AAACCTGTCGTGCCAGCTGCAT	M13
R4-32	5'AAACCTGTCGTGCCAGCTGCATTAATGAATCG	M13
R6-16	5'AAACCTGTCGTGCCAG	M13
RR1	5'AATCAATAATCGGCTGTCTTTCCT	M13
RR2	5' GAA TTT ATC AAA ATC ATA GGT CTG A	M13
N6	5'NNNNNN	
N15	5'NNNNNNNNNNNNNNN	
N21	5'NNNNNNNNNNNNNNNNNNNNN	

Supplemental Table 2. Primer pairs and amplicons

Primer pair name	Primers	target	Amplicon size (bp)
#A	F41+R-43mer	pUC19	277
#9	F40+R39	pUC19	700
#10	F30+R39	pUC19	700
#19	F41+R39	pUC19	1174
#21	F23+R39	pUC19	1174
#24	F23+700R30	pUC19	1174
#25	F1-22 +R1-25	M13	1069
#35	F6-32+R2-31	M13	1610
#37	F4-23+R3-22	M13	2548
#38	F5-27+R3-22	M13	2548
#39	F6-32+R4-32	M13	2548
#40	F7-15+R6-16	M13	2548
#49	60-mer+500-R41	pUC19	974
#57	F23+R3	pUC19	2686
#61	RR1+F4-23	M13	7249
#62	RR2+F1-22	M13	7249

Supplemental Table 3. Compilation of all DNA polymerases tested for gp32-assisted isothermal amplification. Data for strand displacing properties was collected from literature and product information.

DNA Polymerase	Test conc. range (U/μl)	Amplification	Strand displacing
Bsu, LF	0.16-0.48	+	+
Bst 2.0	0.16-0.52	+	+
Bst 3.0	0.16-0.32	+	+
Klenow	0.10-0.33	+	+
Sequenase V2	0.26-0.78	+	+
Phi29	0.26-0.78	-	+
E. coli Pol	0.20-0.40	-	-
T4 DNA polymerase	0.06-0.18	-	-
Vent(exo-)	0.64	-	+
